# Lack of *EGFR*-activating mutations in European patients with triple-negative breast cancer could emphasise geographic and ethnic variations in breast cancer mutation profiles

**DOI:** 10.1186/bcr3079

**Published:** 2011-12-22

**Authors:** William Jacot, Evelyne Lopez-Crapez, Simon Thezenas, Romain Senal, Frédéric Fina, Frédéric Bibeau, Gilles Romieu, Pierre-Jean Lamy

**Affiliations:** 1 Département d'Oncologie Médicale, CRLC Val d'Aurelle-Paul Lamarque, 208, rue des Apothicaires, F-34298 France; 2 Laboratoire de Biologie Spécialisée et d'Oncogénétique, CRLC Val d'Aurelle-Paul Lamarque, 208, rue des Apothicaires, F-34298 France; 3 Département de Biostatistiques, CRLC Val d'Aurelle-Paul Lamarque, 208, rue des Apothicaires, F-34298 France; 4 Laboratoire de Transfert d'Oncologie Biologie, Assistance Publique-Hôpitaux de Marseille, Boulevard Pierre Dramard, Marseille, F-13916, France; 5 Laboratoire de Pathologie, CRLC Val d'Aurelle-Paul Lamarque, 208, rue des Apothicaires, F-34298 France

## Abstract

**Introduction:**

Triple-negative breast cancers (TNBCs) are characterised by lack of expression of hormone receptors and epidermal growth factor receptor 2 (HER-2). As they frequently express epidermal growth factor receptors (EGFRs), anti-EGFR therapies are currently assessed for this breast cancer subtype as an alternative to treatments that target HER-2 or hormone receptors. Recently, *EGFR*-activating mutations have been reported in TNBC specimens in an East Asian population. Because variations in the frequency of *EGFR*-activating mutations in East Asians and other patients with lung cancer have been described, we evaluated the *EGFR *mutational profile in tumour samples from European patients with TNBC.

**Methods:**

We selected from a DNA tumour bank 229 DNA samples isolated from frozen, histologically proven and macrodissected invasive TNBC specimens from European patients. PCR and high-resolution melting (HRM) analyses were used to detect mutations in exons 19 and 21 of *EGFR*. The results were then confirmed by bidirectional sequencing of all samples.

**Results:**

HRM analysis allowed the detection of three *EGFR *exon 21 mutations, but no exon 19 mutations. There was 100% concordance between the HRM and sequencing results. The three patients with *EGFR *exon 21 abnormal HRM profiles harboured the rare R836R SNP, but no *EGFR*-activating mutation was identified.

**Conclusions:**

This study highlights variations in the prevalence of *EGFR *mutations in TNBC. These variations have crucial implications for the design of clinical trials involving anti-EGFR treatments in TNBC and for identifying the potential target population.

## Introduction

Triple-negative breast cancers (TNBCs), which are defined by the lack of oestrogen receptor (ER), progesterone receptor (PR) and human epidermal growth factor receptor 2 (HER-2) expression, account for approximately 15% of all breast carcinomas [1]. TNBCs occur most frequently in young women and tend to have more aggressive, metastatic behaviour and a worse prognosis than other breast cancers. New systemic therapies are urgently needed, as most patients experience TNBC relapse with distant metastases. In addition, hormonal therapies and HER-2-targeted agents are ineffective in this group of patients because of the lack of expression of these therapeutic targets in tumour cells. Currently, chemotherapy is the only systemic therapeutic option for this type of tumour. Despite the recent breakthrough related to the development of poly(ADP-ribose) polymerase inhibitors [2], sustained remission in advanced TNBC is rare and additional targeted therapies are crucially needed.

The receptor tyrosine kinase epidermal growth factor receptor (EGFR) is frequently (30% to 52%) expressed in TNBC [3] and is associated with poor prognosis [4-6]. However, the results of clinical trials on the role of anti-EGFR targeted therapies in this setting remain disappointing [7-11]. Work on the response of non-small-cell lung cancer (NSCLC) to anti-EGFR therapies suggests that EGFR expression detected by immunohistochemistry (IHC) is not the best indicator of tumour-cell dependence on EGFR [12]. Conversely, the presence of *EGFR*-activating mutations is highly predictive of response to treatment with gefitinib or erlotinib [13,14]. These mutations are usually exon 19 deletions and the L858R substitution in exon 21, as well as, rarely, exon 18 mutations. They cluster around the ATP-binding pocket of the tyrosine kinase domain of EGFR, leading to ligand-independent activation of the receptor and longer activation time compared to wild-type EGFR. Exon 20 mutations, however, are associated with resistance to anti-EGFR therapies [13-17].

*EGFR *mutations that may predict sensitivity to EGFR inhibitors have been identified in TNBC as well. Teng and collaborators [18] recently reported that 11.4% of their TNBC series harboured *EGFR *exon 19 or 21 mutations. Because the frequency of *EGFR*-activating mutations in Asian and European patients with NSCLC is quite different [19-21], however, it is important to validate these results in a European population of patients with TNBC before considering the worldwide potential of developing anti-EGFR targeted therapies in TNBC patients.

## Materials and methods

### Patients and tumour samples

A total of 1,695 consecutive patients with breast cancer referred to the Val d'Aurelle Cancer Centre between 2002 and 2010 were prospectively entered into the database of a tumour DNA bank. The stored DNA was isolated (see below) from frozen, histologically proven and macrodissected invasive breast cancer specimens that were handled primarily for ER and PR testing by using the dextran charcoal method as previously described [22,23]. Most of the tumour samples dedicated to molecular analysis were selected on the basis of the immediate diagnosis by using frozen sections. Moreover, additional tumour tissue samples were chosen following the definitive histological diagnosis (with quantification of the percentage of tumour cells) and grade assessment after fixation. This was possible because frozen and formalin-fixed tumour tissue samples were selected from the same tumour areas. Only samples in which at least 50% of tumour cells observed were used for further analysis for *EGFR *mutations. Tumours were considered ER- and PR-positive when the receptor concentration was higher than 10 fM/mg of protein or more than 10% tumour cells were stained by IHC. Patients with primary HER-2-negative breast cancer were initially selected based on the level of HER-2 protein expression by IHC using the A485 monoclonal antibody (Dako A/S, Glostrup, Denmark). HER-2 scores of 0 and 1+ were considered negative. Gene amplification was evaluated using fluorescence *in situ *hybridisation or chromogenic *in situ *hybridisation for HER-2 score 2+ tumours as assessed by IHC. HER2 3+ scores were considered positive. A total of 229 DNA samples from TNBCs (ER-, PR- and HER-2-negative) were ultimately selected for this study. This study was reviewed and approved by our Institutional Review Board. All patients gave their written informed consent.

### DNA extraction

Each frozen tumour specimen was pulverised in liquid nitrogen with an automatic grinder (Cryobroyeur 2000P Automatique; Rivoire, Montpellier, France), homogenised in a Polytron homogeniser (Brinkmann Scientific Instruments, Inc, Westbury, NY, USA) with buffer (20 mM Tris·HCI, 1.5 mM ethylenediaminetetraacetic acid (EDTA), 10 mM Na_2_MoO_4_, 1.5 mM dithiothreitol, 10% glycerol, pH 7.4) (buffer-to-tissue ratio 10:1 (vol/wt)) and centrifuged at 10,000 × *g *for 15 minutes. Total genomic DNA was extracted from the pellet using the QIAamp DNA Mini Kit (51304; QIAGEN, Hilden, Germany) according to the manufacturer's protocol. DNA yield and purity were quantitated and assessed by measuring the absorbance at 260 nm and 280 nm with a NanoDrop spectrophotometer (Thermo Fisher Scientific, Waltham, MA, USA). All samples had a 260-nm to 280-nm ratio higher than 1.7. DNA was stored at -20°C in Tris-EDTA buffer (10 mM Tris and 0.5 mM EDTA, pH 7.6).

### PCR amplification and high-resolution melting analysis

PCR amplification and high-resolution melting (HRM) analysis were performed on a Rotor-Gene 6000™ (Corbett Life Science, Mortlake, NSW, Australia) using the LightCycler 480 High Resolution Melting PCR Master Mix kit (04 909 631 001; Roche Diagnostics, Meylan, France). Primers were designed to amplify *EGFR *fragments that span the region in which the exon 19 deletions (182-bp amplicon) and the L858R mutation in exon 21 (164-bp amplicon) are localised (Table 1). PCR amplifications were performed in a final volume of 20 μl that included 10 ng of purified genomic DNA, 10 μl of 2 × PCR mix, 2.4 μl of 25 mM MgCl_2 _and 0.4 μl of each 10 μM forward and reverse primer. The following cycling conditions were used: one cycle of 95°C for 5 minutes; then 50 cycles of 95°C for 15 seconds, 63°C to 58°C for 15 seconds (with a decrease in annealing temperature of 0.5°C per cycle during the first 11 cycles), 72°C for 20 seconds; then one cycle of 95°C for 1 minute, 40°C for 1 minute, 65°C for 20 seconds, and finally a melt from 65°C to 95°C rising at a rate of 0.1°C per 2 seconds. The melting conditions included one cycle at 95°C for 1 minute, one cycle at 40°C for 1 minute and one cycle at 65°C for 2 seconds, followed by a melt from 65°C to 95°C rising at a rate of 0.1°C/second. HRM data were analysed using Rotor-Gene 6000 version 1.7 software (Corbett Life Science). For each sample, the normalised melting curves were evaluated and samples were compared with a control consisting of genomic DNA from the LNCaP cell line (wild-type *EGFR*) in a deduced difference plot. Significant deviations from the horizontal line relative to the spread of the wild-type control were indicative of sequence changes within the analysed amplicon. Genomic DNA from SW620 cells was used as a second wild-type *EGFR *control, whereas DNA from the H1650 and H1975 human NSCLC cell lines was used as positive controls for *EGFR *exon 19 and exon 21 heterozygous mutations, respectively. Water was used as a negative control for PCR contamination.

**Table 1:** Primer sequences^a^

GenBank accession number	Gene name and primer	Sequence 5'-3'	Size (nt)	GC (%)	Amplicon (bp)	Annealing temperature
NM_005228	*EGFR* Exon 19 forward	CGTCTTCCTTCTCTCTCTGTCATAGG	26	50%	182	58°C
	*EGFR* Exon 19 reverse	AAAAGGTGGGCCTGAGGTTC	20	55%		
	*EGFR* Exon 21 forward	TTGGAGGACCGTCGCTTG	18	61.1%	164	58°C
	*EGFR* Exon 21 reverse	ACCTAAAGCCACCTCCTTACTTTG	24	45.8%		

^a^EGFR = epidermal growth factor receptor; GC = percentage of guanines and cytosines in the primers' sequence; nt = nucleotide.

### DNA sequencing

After HRM analysis, PCR products were purified using the ExoSAP-IT PCR Purification Kit (US78200; GE Healthcare, Saclay, France) according to the manufacturer's instructions. Purified PCR products were then used as templates for sequencing with the BigDye Terminator version 1.1 Cycle Sequencing Kit (4336774; Applied Biosystems, Inc, Foster City, CA, USA). Reactions were run on a Mastercycler gradient thermal cycler (Eppendorf, Lepecq, France) according to the following protocol: one cycle of 96°C for 2 minutes; then 25 cycles of 96°C for 10 seconds, 50°C for 5 seconds and 60°C for 2 minutes. 50°C for 5 seconds and 60°C for 2 minutes. Purification was carried out using the BigDye XTerminator Purification Kit (4376486; Applied Biosystems, Inc). For all samples, bidirectional sequencing was performed by using PCR primers and an Applied Biosystems 3730xl DNA Analyzer. After migration completion, the *EGFR *sequences were analysed using Applied Biosystems Sequencing Analysis version 5.2 software. Genomic DNA from LNCaP and SW620 cells (wild-type *EGFR*) was used as a negative control and genomic DNA from H1650 and H1975 cells was used as a positive control for exon 19 (c.2235_2249del15, p.E746_A750del) and exon 21 (c.2573T > G, p.L758R) mutations, respectively.

## Results

### Patient and tumour characteristics

A total of 229 DNA samples from TNBC tissue specimens that contained a high percentage of tumour tissue as required for ER and PR testing were selected for this study. The main clinicopathological characteristics of the patients with TNBC are summarised in Table 2. The patients' median age was 58 years (range, 29 to 84 years). Ductal carcinoma was the prevalent histological type (79%). Eighteen patients were noted as no clinical data regarding nodal metastatic spreading because the sample used for DNA extraction was obtained during the surgical removal of a local recurrence in individuals who had had a previous lymphadenectomy (14 cases) or a previous metastasectomy for distant recurrence (4 cases). Only nine tumours (4%) were classified as Scarff-Bloom-Richardson grade 1.

**Table 2:** Patient and tumour characteristics^a^

Patient and tumour characteristics	Number of patients (%) (*N *= 229)	Patient age
Median age at diagnosis, years [range]		58 [29 to 84]
Tumour classification		
T1	88 (38)	
T2	109 (48)	
T3	9 (4)	
T4	21 (9)	
TX	2 (1)	
Node classification		
N0	136 (59)	
N1	47 (21)	
N2	20 (9)	
N3	8 (3)	
NX	18 (8)	
Metastasis classification		
M0	212 (92)	
M1	13 (6)	
MX	4 (2)	
Histology		
Ductal	180 (79)	
Lobular	14 (6)	
Other^b^	35 (15)	
SBR grade		
I	9 (4)	
II	70 (31)	
III	146 (64)	
NE	4 (2)	
Tubule formation		
1	5 (2)	
2	31 (14)	
3	178 (78)	
NE	15 (6.55)	
Nuclear pleomorphism		
1	5 (20)	
2	66 (29)	
3	102 (44)	
NE	16 (7)	
Mitotic count		
1	45 (20)	
2	66 (29)	
3	102 (44)	
NE	16 (7)	
Peritumoural vascular invasion		
Yes	115 (50)	
No	53 (23)	
NE	61 (27)	

^a^MX = no clinical data regarding metastatic spreading; NE = not evaluated; NX = no clinical data regarding nodal status; TX = no clinical data regarding tumour size. ^b^Nine undifferentiated carcinomas, six invasive papillary carcinomas, four mixed ductal-lobular carcinomas, three apocrine carcinomas, four medullary carcinomas, three metaplastic carcinomas, two sarcomatoid carcinomas, two adenoid cystic carcinomas, and one case each of the following histological subtypes: adenosquamous and neuroendocrine.

### Assay validation and sensitivity testing

**Figure 1:**
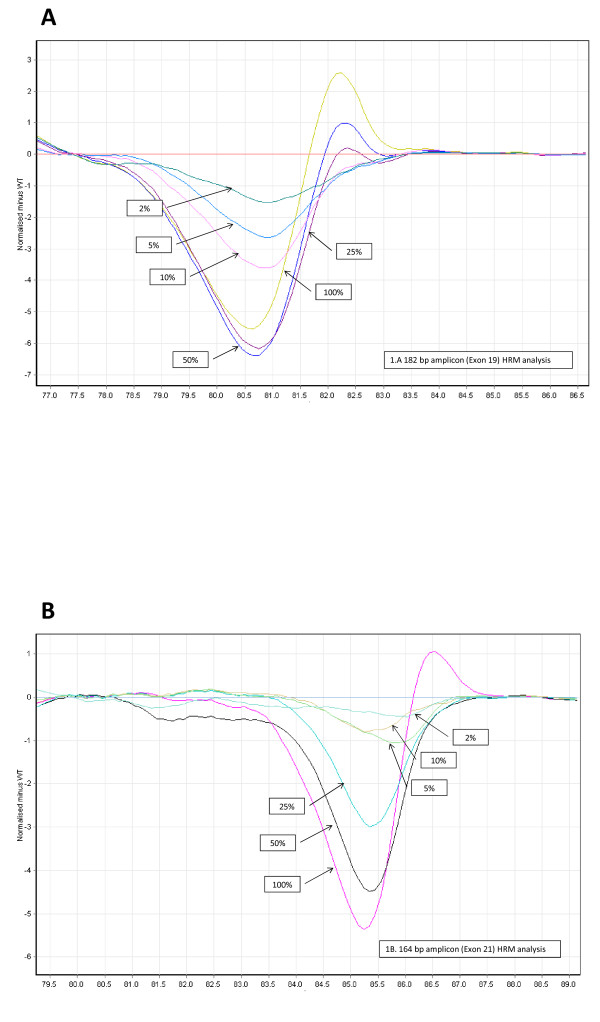
**Sensitivity for the *EGFR *high-resolution melting assays**. Serial dilutions of mutant DNA in proportions of 50%, 25%, 10%, 5% and 2% were compared with the wild-type control sample (LNCaP cell DNA) to determine the sensitivity of the test. **(A) **Serial dilutions of genomic DNA from the H1650 cell line were compared with the control sample to produce the difference plot for the 182-bp (exon 19) amplicon. **(B) **Serial dilutions of genomic DNA from the H1975 cell line were compared with the control sample to produce the difference plot for the 164-bp (exon 21) amplicon. HRM = high-resolution melting.

First, genomic DNA from the H1650 and H1975 NSCLC cell lines with known *EGFR *mutations was used to test the HRM mutation detection method. In the deduced difference plot, the heteroduplex melting patterns of the two cell lines were unambiguously different from the curves of control LNCaP and SW620 cells (wild-type *EGFR*). The two wild-type controls had comparable HRM curves (greater than 99% homology). The sensitivity of the HRM mutation detection method with the *EGFR *exon 19 and exon 21 primer pairs was tested by using serial dilutions of genomic DNA from H1650 (exon 19 deletion) and H1975 (L858R substitution in exon 21) cells in wild-type DNA to obtain proportions of 50%, 25%, 10%, 5% and 2% of mutant DNA. For both primer pairs, 5% of mutant DNA in the template was the lower limit of detection (Figure 1), indicating that tumour samples containing at least 5% tumour cells could be reliably screened by this method. This level of sensitivity is in agreement with previously reported HRM data [24].

### *EGFR *mutation detection by high-resolution melting analysis and sequencing in genomic DNA from TNBC samples

The EGFR exon 19 (182-bp amplicon) and exon 21 (164-bp amplicon) primer pairs were used to screen the 229 TNBC DNA samples. All samples were successfully amplified. To confirm the quality of the DNA (previously controlled by spectrometry), the number of cycles needed to reach the fluorescence threshold (that is, the cycle threshold (Ct) value) was measured. The mean Ct value was 23.0 cycles (range, 18.9 to 25.7 cycles) for the 182-bp amplicon and 22.8 cycles (range, 19.8 to 25.3 cycles) for the 164-bp amplicon. The absence of poor or late amplification confirmed the quality of the DNA samples. The HRM curve analysis allowed the identification of three abnormal profiles for the EGFR exon 21 amplicon and none for the EGFR exon 19 amplicon.

Bidirectional sequencing analysis was performed for all 229 DNA samples and confirmed the HRM results. A synonymous SNP in EGFR exon 21 (c.2508C > T, p.R836R) that corresponded to the SNP rs17290559 in the National Center for Biotechnology Information (NCBI) SNP database (National Institutes of Health, Bethesda, MD, USA) was identified in the three samples with altered HRM profiles. The 226 samples with HRM profiles similar to those of wild-type controls were confirmed as having wild-type EGFR by sequencing analysis. No *EGFR*-activating mutation was found.

## Discussion

Clinical studies are currently underway to evaluate the efficacy of EGFR tyrosine kinase inhibitors in patients with TNBC, because hormonal therapies and HER-2-targeted agents are ineffective. The evaluation of the correlation between *EGFR*-activating mutations and the response of TNBC to EGFR targeted therapies, as well as the determination of the percentage of patients who might harbour an *EGFR*-activating mutation in tumour cells, are essential prerequisites for comprehensive clinical development of EGFR tyrosine kinase inhibitor trials in this setting.

Herein we report the results of *EGFR *mutational analysis of a large, comprehensive set of 229 European TNBC patients by HRM analysis and bidirectional sequencing. To the best of our knowledge, this is the largest cohort of patients with TNBC in whom the presence of *EGFR*-activating mutations has been investigated. We found no activating mutations (exon 19 deletions or the L858R substitution) in our 229 samples. HRM is a high-throughput method for genotyping and mutation scanning [25,26]. It allows detection of mutations related to base substitution or base deletions with high sensitivity and high specificity. The sensitivity and specificity of HRM analysis is higher with DNA isolated from frozen samples [27], and HRM results are also accurate with regard to samples harbouring low-prevalence mutations [24,28]. The Ct values obtained during the real-time PCR used for HRM assay confirmed the quality of our DNA samples that were extracted from freshly frozen tumours. One limitation of the HRM assay is that the melting profiles of samples carrying deletions do not show the same magnitude of change observed with DNA harbouring heterozygous SNPs [29]. Moreover, depending on the SNP class, the melting curve shifts are more or less important. Nevertheless, we observed no discordance between HRM and sequencing results. The C > T base change at codon 836 of EGFR exon 21 is a class 1 SNP and was unambiguously detected by HRM in three samples, whereas no T > G substitution (class 2 SNP), which corresponds to the *EGFR *L858R activating mutation, was detected. The percentage of heterozygous genotypes in our TNBC series is in agreement with the data in the NCBI SNP database for the HapMap-CEU cohort (3.4%) and the exome sequencing project cohort (2.8%). If we consider sequencing the gold standard, our HRM test for detection of *EGFR *mutations in exons 19 and 21 had 100% sensitivity and specificity. Although sequencing analysis cannot be considered 100% accurate, owing to the low sensitivity of direct sequencing [30,31], the high tumour cellularity after macrodissection, as well as the quality of the DNA isolated from our frozen TNBC samples, allow us to be confident in our results and to conclude that our TNBC samples did not present *EGFR*-activating mutations that are detectable using the routine techniques used in molecular biology laboratories. A variation in the percentage of tumour cells in the samples could not explain the lack of detection of *EGFR *mutations in our series based on the 5% sensitivity threshold of our HRM test.

These results differ strikingly from those of the study by Teng *et al*. [18], who recently reported the presence of *EGFR*-activating mutations, particularly exon 19 deletions and exon 21 missense (L858R) mutations, in 11.4% of 70 TNBC samples from a cohort of 653 patients with TNBC (the cutoff value for ER and PR positivity was 10% like in our study). Moreover, this frequency of *EGFR*-activating mutations was higher than that described in previous work. Bhargava *et al*. reported no *EGFR *exon 19 in-frame deletions or exon 21 L858R point mutations in 11 *EGFR*-amplified sporadic breast tumours, of which 8 were TNBC [32]. In the study by Reis-Filho *et al*., no *EGFR*-activating mutation was identified in a group of 47 metaplastic breast carcinomas, a subset of basal-like breast cancers in which EGFR is overexpressed in 80% of the cases and amplified in about one third of them [6]. Generali *et al*. identified only *EGFR *silent mutations in 42 unselected sporadic breast tumours [33]. Toyama *et al*. analysed 58 tumour samples from Japanese patients with TNBC to detect 14 known *EGFR *mutations, including exon 19 deletions and the exon 21 L858R mutation, but none was found [34]. Lv *et al*. recently reported the presence of two (1.4%) EGFR activating mutations in 139 Chinese women with breast cancers (10 triple-negative tumours) using RT-PCR (1 exon 19 deletion and 1 exon 21 L858R mutation) [35]. The two mutations were in triple-negative ductal carcinoma and in ER-positive ductal carcinoma. A higher rate of *EGFR *missense mutations (45.8%) was reported in hereditary BRCA1/2 positive breast tumours, which are typically triple-negative, than in sporadic breast cancers (14.6%) [36]. However, these were often silent rather than activating mutations.

Lamy *et al*. recently reported 4.7% artefactual mutations in *KRAS *using formalin-fixed paraffin-embedded colon cancer tissue samples [37]. As noted by Marchetti *et al*., "These artifacts can easily be observed by carrying out multiple PCR amplifications of very small amounts of DNA, particularly if the DNA is isolated from paraffin-embedded tissues" [38] (p. 526). This could be another explanation for the discrepancies of the results of the different studies and raises the question of the impact of the processing methods on the outcomes of mutational analyses. However, it is important to consider that these artefacts concerned C→T/G→A substitutions and not exon 19 deletions or exon 21 L858R mutations. Thus it is unlikely that fixation artefacts might entirely explain the different percentages of *EGFR *mutations reported by the aforementioned researchers.

On the other hand, the different frequencies of *EGFR*-activating mutations reported by Teng *et al*. [18], in our present study and by other researchers could suggest, as already clearly shown for NSCLC, geographic and ethnic variations in the frequency of *EGFR*-activating mutations [21,39,40]. Indeed, the population studied by Teng *et al*. was predominantly Chinese, whereas the other studies were focused mainly on Japanese, American or European cohorts. The identification of geographic and/or ethnic variations in the frequency of *EGFR*-activating mutations in two distinct tumour types could also suggest the possible existence of specific environmental carcinogenic factors that might play a role in NSCLC and TNBC development and/or progression in East Asian patients.

## Conclusions

Herein we report *EGFR *mutational analysis in a set of 229 European patients with TNBC. To our knowledge, this is the largest cohort of patients with TNBC in whom the presence of *EGFR*-activating mutations has been investigated. We found no activating mutation (exon 19 deletion or L858R mutations) in our 229 tumour samples. The striking difference in mutational frequency between our study (0%) and the work by Teng *et al*. [18] (11.4%) could highlight the existence of geographic and ethnic variations in the prevalence of *EGFR *mutations in TNBC. These variations have crucial implications for the design of clinical trials involving anti-EGFR therapies in patients with TNBC, particularly for the selection of the potential target population.

## Abbreviations

bp: base pair; BRCA1: breast cancer type 1 susceptibility protein; Ct: cycle threshold; EDTA: ethylenediaminetetraacetic acid; EGFR: epidermal growth factor receptor; ER: oestrogen receptor; HER-2: epidermal growth factor receptor 2; HRM: high-resolution melting; IHC: immunohistochemistry; MX: no clinical data regarding metastatic spreading; NE: not evaluated; NSCLC: non-small-cell lung cancer; PCR: polymerase chain reaction; PR: progesterone receptor; RT: reverse transcriptase; SNP: single-nucleotide polymorphism; TNBC: triple-negative breast cancer; TX: no clinical data regarding tumour size.

## Competing interests

The authors declare that they have no competing interests.

## Authors' contributions

WJ contributed to the conception and design of the entire study, selected the eligible patients, acquired the clinicopathological data and contributed to the drafting of the manuscript. EC was responsible for designing and optimising the HRM analysis and contributed to data interpretation and the critical revision of the manuscript. ST supervised the statistical analysis and assisted in drafting the manuscript. RS carried out most of the experiments on DNA samples. FF designed the oligonucleotide primers and contributed to the critical revision of the manuscript. FB performed tumour analysis and contributed to the critical revision of the manuscript. GR made the original observations leading to this work and contributed to the critical revision of the manuscript. PJL contributed to the conception and design of the entire study, coordinated sample collection, interpreted data and contributed to the drafting of the manuscript. All authors read and approved the final manuscript.
